# Association of Sex, Age, and Inflammatory Cell Counts with Complicated Acute Appendicitis

**DOI:** 10.3390/pathophysiology33010022

**Published:** 2026-03-14

**Authors:** Said José Serrano Guzmán, Carlos Leyber Vargas Juárez, Marcos Hernández Gómez, José Roberto Luis Vásquez, Sergio Roberto Aguilar Ruiz, Juan Carlos Ramos Martínez, Joscelin Amaranta Macías Ríos, Edgar Gustavo Ramos Martínez, José Luis Cano Pérez, Jesús David Guzmán Ortiz, Martha Silvia Martínez Luna, Leticia Lorena Hernández González

**Affiliations:** 1 División de Cirugía, Hospital Básico Comunitario de Río Grande Villa de Tututepec, Instituto Mexicano del Seguro Social para el Bienestar, Oaxaca 71830, Mexico; 2 Facultad de Sistemas Biológicos e Innovación Tecnológica, Universidad Autónoma “Benito Juárez” de Oaxaca, Oaxaca 68120, Mexicojcano@uabjo.mx (J.L.C.P.); 3 División de Cirugía, Hospital Cruz Azul, Oaxaca 70380, Mexico; 4 División de Cirugía, Hospital General San Pedro Pochutla, Oaxaca 70900, Mexico; 5 Facultad de Medicina y Cirugía, Universidad Autónoma “Benito Juárez” de Oaxaca, Oaxaca 68120, Mexicoedgargus2@gmail.com (E.G.R.M.); 6 Unidad Médica de Alta Especialidad UMAE Yucatán, Ignacio García Téllez, Yucatán 97155, Mexico; 7 División de Medicina Interna, Hospital General “Dr. Aurelio Valdivieso”, Instituto Mexicano del Seguro Social Para el Bienestar, Oaxaca 68050, Mexico; 8 División de Cirugía, Hospital General “Dr. Aurelio Valdivieso”, Instituto Mexicano del Seguro Social Para el Bienestar, Oaxaca 68050, Mexico

**Keywords:** acute appendicitis, complicated appendicitis, neutrophils, sex factors, risk factors, logistic regression models, marginal effects

## Abstract

Background/Objectives: Sex and age influence inflammatory responses, but researchers have not fully characterized their combined association with complicated acute appendicitis (CAA). This study assessed the independent and interactive associations of sex, age, and inflammatory cell counts with CAA. Methods: We conducted a retrospective observational study of 708 patients with histopathologically confirmed uncomplicated appendicitis (UAA) or CAA. We analyzed demographic and clinical data, including preoperative complete blood counts, stratified by sex. We used multivariable logistic regression models with interaction terms to evaluate associations and possible effect modification by sex and age. We explored the direction and magnitude of these interactions by estimating marginal predicted probabilities. Results: The incidence of CAA was significantly higher in men than in women. In men with CAA, complete blood count analysis showed elevated neutrophil and monocyte counts and reduced lymphocyte counts. Male sex (odds ratio (OR) 2.197, 95% confidence interval (CI) 1.610–2.999), continuous age (1.017, 1.002–1.033), lymphocyte count (0.656, 0.526–0.820), monocyte count (1.551, 1.036–2.321), and platelet count (1.004, 1.001–1.006) were independently associated with CAA. Interaction analysis revealed significant interactions between neutrophils and both sex and age (*p* < 0.05), while lymphocyte counts showed significant interaction with age but not with sex. Conclusions: This study provides new insight into complex sex- and age-related immune cell patterns in CAA and may inform future diagnostic and management strategies by highlighting immune profile variability.

## 1. Introduction

Acute appendicitis (AA) is the sudden inflammation of the vermiform appendix and constitutes the most common cause of abdominal surgical emergencies worldwide [1]. The global incidence is 214 cases per 100,000 individuals annually, accounting for nearly 17 million new cases each year [2]. The primary etiology involves obstruction of the appendiceal lumen, leading to ischemia, bacterial proliferation, activation, and infiltration of innate immune cells. The magnitude and persistence of this inflammatory response play a central role in the progression to complicated forms of disease, which can culminate in tissue necrosis, gangrene, and perforation [3]. This severe presentation is classified as complicated acute appendicitis (CAA), encompassing gangrenous and perforated forms. In contrast, in the absence of these complications, the condition is defined as uncomplicated acute appendicitis (UAA), which includes catarrhal and phlegmonous appendicitis [4]. Appendiceal perforation can result in peritonitis or sepsis, and is associated with increased morbidity and mortality, elevated surgical risk, prolonged hospitalization, and higher healthcare costs [5].

AA and CAA have historically shown sex-related differences in incidence, clinical presentation, diagnostic methods, and outcomes. However, these disparities have not been thoroughly examined in the literature [6,7,8]. Many studies show that men have AA more often and are at higher risk of CAA [9,10]. By contrast, women more frequently experience diagnostic delays, more negative appendectomies, and less postoperative pain [11,12,13]. Still, the biological reasons for these differences are not well understood or investigated.

Sex-related variation in immune and inflammatory responses may explain differences in AA outcomes. For example, hormonal factors might modulate inflammatory pathways, as suggested by the reduced incidence of appendicitis during pregnancy—a possible immunomodulatory effect [14]. Growing evidence indicates that men and women exhibit distinct immune responses to inflammatory and infectious diseases, including COVID-19, pancreatitis, and autoimmune diseases [15,16,17,18]. Nevertheless, data on sex-based differences in inflammatory cell profiles in acute appendicitis are limited [7].

Age is another significant factor in immune response. As people grow older, their immune systems change, with alterations in the number and function of immune cells that affect innate and adaptive immunity, which may influence how AA progresses [19,20,21]. Older adults also tend to show different patterns of inflammation to younger age groups [9,22].

Diagnosing AA or CAA requires clinical findings, imaging, and inflammation markers such as C-reactive protein and immune cell counts, which are often used in scoring systems [5,23,24,25]. Many biochemical markers have been suggested for CAA, but the complete blood count is a more cost-effective and widely used tool in emergencies. It indirectly measures inflammation by counting neutrophils, lymphocytes, monocytes, and platelets [26].

Despite the widespread use of immune cell counts as markers of systemic inflammation, evidence regarding their association with CAA according to sex and age remains limited [6,7]. Therefore, this study aimed to examine the associations between inflammatory cell counts, sex, and age in relation to CAA. Specifically, the objectives were to: (1) evaluate associations of sex, age, and blood cell counts with CAA probability; (2) assess heterogeneity through interaction terms, namely, age × sex, age × cell counts, and sex × cell counts; and (3) describe the direction and magnitude of these interactions in relation to CAA.

## 2. Materials and Methods

### 2.1. Participants, Variables, and Study Design

A retrospective observational study compared clinical, demographic, and laboratory variables between men and women diagnosed with complicated acute appendicitis (CAA) and uncomplicated acute appendicitis (UAA). Medical records were reviewed for patients treated in the General Surgery Department of Hospital Dr. Aurelio Valdivieso, Oaxaca, Mexico, from July 2020 to July 2024. The study analyzed clinical records from 708 patients with acute appendicitis: 350 women and 358 men. The protocol received Institutional Research Committee approval (approval number: HGDAV/CI/0002/2024).

The inclusion criteria comprised male and female patients older than 15 years with a histopathological diagnosis of acute appendicitis. All variables of interest were routinely obtained as part of mandatory preoperative clinical and laboratory assessments. Complete data were available for all included patients. Records were excluded if they pertained to pregnant patients or individuals with immunodeficiencies, active viral or bacterial infections, recent transfusions, or a diagnosis other than acute appendicitis, such as gynecological conditions (ovarian cysts, salpingitis, or tubal abscess) or diverticulitis. These exclusions reflect alternative causes of acute abdominal pain and were applied to ensure diagnostic homogeneity rather than representing sex-based selection bias. CAA was defined by the presence of gangrenous or perforated findings during surgery. UAA was defined as cases lacking perforation or necrosis.

Data were collected on demographic factors (sex, age), medication use (antibiotics, non-prescription anti-inflammatory drugs), and comorbidities (diabetes, hypertension, obesity). Clinical signs and symptoms included anorexia, pain migration, fever over 38 °C, nausea or vomiting, right lower quadrant pain, rebound tenderness in the right iliac fossa, and muscular guarding/Blumberg’s sign. Laboratory parameters measured were leukocyte, neutrophil, monocyte, and platelet count; mean platelet volume; activated partial thromboplastin time (aPTT); prothrombin time (PT); and international normalized ratio (INR). Ultrasonographic evaluation identified appendicolith, periappendicular plastron, abscess, and free fluid in the periappendiceal cavity. Additionally, the neutrophil-to-lymphocyte ratio (NLR), platelet-to-lymphocyte ratio (PLR), monocyte-to-lymphocyte ratio (MLR), and neutrophil × platelet/lymphocyte ratio (systemic inflammation index, SII) were recorded.

### 2.2. Variable Processing

Data were entered into an electronic spreadsheet for analysis. Continuous variables were analyzed as recorded. Categorical variables were coded as 1 for present and 0 for absent, with male sex as 1 and female sex as 0. These variables, corresponding to mandatory preoperative clinical and laboratory assessments for all patients with confirmed acute appendicitis at our institution, were systematically recorded. The analytical dataset contained no missing values for the variables included in the models. Therefore, no imputation procedures were required. To facilitate clinical interpretation and graphical presentation, patients were stratified into four groups based on recognized differences in immune function across the lifespan, as supported by prior evidence. The groups were defined as follows: <20 years, representing childhood/adolescence and a period of ongoing immune maturation; 20–39 years, indicating young adulthood with relatively stable immunity; 40–59 years, reflecting midlife when early age-related immune changes occur; and ≥60 years, capturing older adulthood during which immunosenescence tends to be established [27,28,29,30,31].

For inferential analyses, age was treated as a continuous variable to preserve statistical power and prevent information loss due to categorization. However, to facilitate interpretation and reduce multicollinearity and instability in the interaction models, age was mean-centered (mean = 32.6 years).

### 2.3. Statistical Analysis

Descriptive analyses were stratified by sex. Continuous variables are reported as means with standard deviations or as medians with interquartile ranges, depending on their distribution, determined using the Kolmogorov–Smirnov test. Categorical variables are presented as frequencies and percentages. To assess group differences, Student’s *t*-test was used for normally distributed continuous variables, and the Mann–Whitney U test for non-normally distributed variables. Frequency comparisons were conducted using chi-square tests. To control the error from multiple comparisons, the Benjamini–Hochberg procedure was applied to limit the false discovery rate (FDR). All descriptive analyses were performed using GraphPad Prism version 8.0.2 (Boston, MA, USA). Figures were generated using both Prism and R version 4.3.1 (R Foundation for Statistical Computing, Vienna, Austria).

### 2.4. Logistic Regression Analysis

Associations between sex, age, and immune cell counts and complicated acute appendicitis were estimated using binary logistic regression models. CAA was the dependent variable, coded 1, and UAA was coded 0.

Bivariate logistic regression model

$$log\left(\frac{p}{1-p}\right)=\beta_{0}+\beta_{1}X_{1}$$

where *p* represents the probability of CAA, $\beta_{0}$ is the intercept, $\beta_{1}$ is the regression coefficient associated with the independent variable and $X_{1}$. 

Multivariable logistic regression model

$$log\left(\frac{p}{1-p}\right)=\beta_{0}+\beta_{1}X_{1}+\beta_{2}X_{2}$$

Multivariable logistic regression model with interaction terms

To evaluate potential effect modification by sex and age, interaction terms were incorporated into multivariable models. The interaction model was specified as:

$$log\left(\frac{p}{1-p}\right)=\beta_{0}+\beta_{1}X_{1}+\beta_{2}X_{2}+\beta_{3}\left(X_{1}\times X_{2}\right)$$

where $X_{1}$ and $X_{2}$ represent the interacting variables, and $X_{1}\times X_{2}$ is the interaction term. In models including interaction terms, β_1_ and β_2_ represent the conditional effects of each variable when the interacting variable is equal to zero, while β_3_ quantifies the departure from additivity on the log-odds scale.

Specifically, the following interaction models were evaluated:

Sex × Age interaction:

$$log\left(\frac{p}{1-p}\right)=\beta_{0}+\beta_{1}X_{Sex}+\beta_{2}X_{Age}+\beta_{3}\left(X_{Sex}\times X_{Age}\right)+\beta_{4}X_{Diabetes}+\beta_{5}X_{Obesity}+\beta_{6}X_{Hypertension}$$

Sex × Inflammatory Cell Count interaction:

$$log\left(\frac{p}{1-p}\right)=\beta_{0}+\beta_{1}X_{Sex}+\beta_{2}X_{Neutrophil\;count}+\beta_{3}\left(X_{Sex}\times X_{Neutrophil\;count}\right)\;\;+\beta_{4}X_{Diabetes}+\beta_{5}X_{Obesity}+\beta_{6}X_{Hypertension}+\;\beta_{7}X_{Age}$$

Age × Inflammatory Cell Count interaction:

$$log\left(\frac{p}{1-p}\right)=\beta_{0}+\beta_{1}X_{Age}+\beta_{2}X_{Neutrophil\;count}+\beta_{3}\left(X_{Age}\times X_{Neutrophil\;count}\right)\;\;+\beta_{4}X_{Diabetes}+\beta_{5}X_{Obesity}+\beta_{6}X_{Hypertension}+\;\beta_{7}X_{Sex}$$

Similar multivariable models were fitted separately for each inflammatory cell count (lymphocytes, monocytes, or platelets), with one cell type included per model, to reduce the risk of model overfitting. All analyses were adjusted for sex, age, and relevant comorbidities, which were included as covariates to minimize confounding bias.

To characterize the direction and magnitude of interactions, we used model-estimated marginal predicted probabilities derived from multivariable logistic regression models. These models included interaction terms and were adjusted for sex, age, and comorbidities, with age centered in all analyses. Predicted probabilities were calculated across the observed range of cell counts and presented graphically, stratified by sex or age group as appropriate. Results are reported as odds ratios with 95% confidence intervals. All analyses were performed using IBM SPSS Statistics version 26.0 (Armonk, NY, USA) and R version 4.3.1.

## 3. Results

### 3.1. A Higher Frequency of Complicated Acute Appendicitis Was Observed in Men than in Women

**Figure 1:**
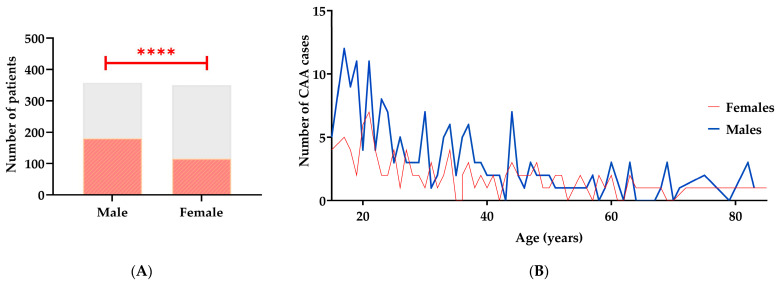
Sex- and age-based distribution of patients with acute appendicitis and CAA. (**A**) Total patients by sex. Gray bars show the number of acute appendicitis cases in males and females. Orange diagonally striped bars mark CAA cases. The sex difference is statistically significant (****, *p* < 0.0001). (**B**) Age distribution of CAA by sex. Lines show the absolute number of cases per year of age. The *y*-axis represents annual counts, not cumulative frequencies. The blue line is for males; the red is for females. Both groups peak at younger ages and decline with age.

Between 2020 and 2024, 744 patients who underwent appendectomy were identified. Of these, 36 were excluded due to postoperative findings unrelated to acute appendicitis, mainly gynecological conditions such as ovarian cysts, salpingitis, tubal abscess, and diverticulitis (Supplementary Table S1). Among the analyzed records, 350 were women and 358 were men. Only one patient had laparoscopic surgery; all others had open procedures. No in-hospital mortality was observed in the study population. The findings showed that, among patients with acute appendicitis, men had a higher frequency of complicated acute appendicitis (CAA) (50.56%, *n* = 181) than women (33.14%, *n* = 116), *p* < 0.0001 (Figure 1A). These sex-related differences in CAA persisted across all age groups (Figure 1B).

### 3.2. The Complete Blood Count Profiles Differ Between Men and Women with CAA

After establishing that male sex and age are associated with CAA, the subsequent objective was to identify preoperative factors displaying different patterns between men and women in both CAA and UAA (Table 1). First, the results showed that CAA was observed more frequently in men than in women across age groups. Next, men with UAA also reported a higher frequency of self-medication with antibiotics. In contrast, no differences were observed between men and women regarding the frequency of comorbidities, clinical signs, or symptoms. Turning to laboratory values, the analysis showed sex-related differences in inflammatory cell counts. Men presented higher median counts of neutrophils, monocytes, NLR, MLR, PLR, and SII than women. They also had lower lymphocyte and platelet counts. No differences were observed between women and men in ultrasound findings. Collectively, these findings describe sex-specific differences in inflammatory cell count profiles among patients with acute appendicitis, particularly in those with CAA.

### 3.3. Male Sex and Age Are Independently Associated with the Occurrence of Complicated Acute Appendicitis

The association between gender, age, and CAA was evaluated using several statistical models. In the bivariate analysis (model 1), male sex and age were significantly associated with CAA (Table 2). The multivariate model (model 2), which adjusted for diabetes, hypertension, and obesity, found that male sex and age remained independently associated with CAA. The interaction between male sex and age was not significant (*p* = 0.318), indicating that the association between age and CAA did not differ by sex (Table 2). In conclusion, these results indicate an association between male sex and CAA across age groups, but no significant interaction between sex and age was observed.

### 3.4. Lymphocytes, Monocytes, and Platelets Are Associated with Complicated Appendicitis

Preoperative studies provided immune cell concentration data, which were analyzed for their association with CAA. Three progressively adjusted logistic regression models were constructed: Model 1, unadjusted; Model 2, adjusted for sex and age; and Model 3, fully adjusted for metabolic comorbidities, including diabetes, hypertension, and obesity. Across all three models, higher monocyte and platelet concentrations, along with lower lymphocyte concentrations, were associated with CAA (Table 3). Notably, neutrophil concentration was not associated with CAA. These analyses showed that immune cell concentrations remained significantly associated with CAA after adjustment for sex, age, and metabolic comorbidities.

### 3.5. Interactions Among Sex, Age, and Immune Cell Profiles in Complicated Acute Appendicitis

To assess whether age or biological sex modified the association between immune cell counts and complicated acute appendicitis, interaction terms for immune cell–age and immune cell–sex were included in binary logistic regression models. Both unadjusted (Supplementary Table S2) and fully adjusted models accounted for clinical covariates and metabolic comorbidities (Table 4). The *p*-values indicate the significance of these interactions. Significant interactions were observed between neutrophil count and age (*p* = 0.008), as well as between neutrophil count and biological sex (*p* = 0.002). In contrast, lymphocyte count did not significantly interact with biological sex; however, a significant and consistent interaction between lymphocyte count and age was detected (*p* = 0.002) (Table 4). No significant interactions were identified between monocyte or platelet counts and either age or biological sex.

**Figure 2:**
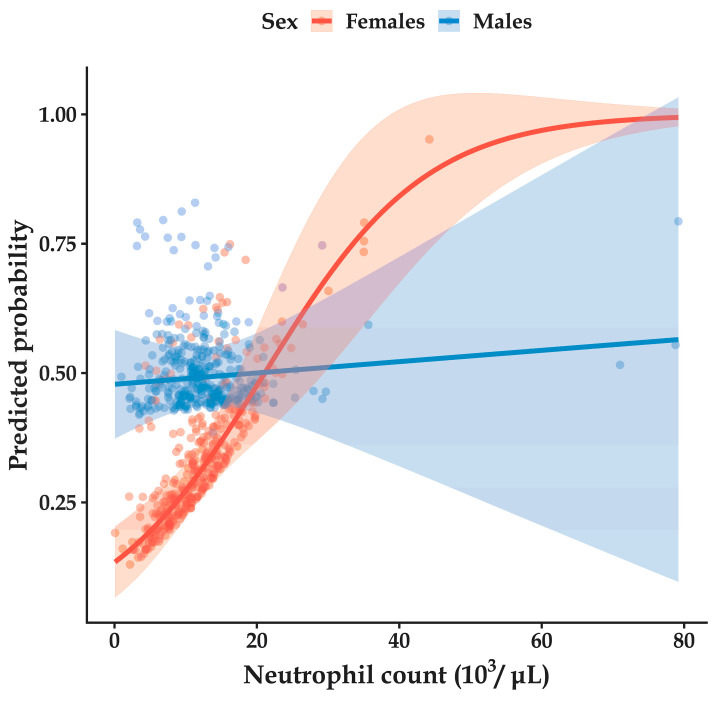
Adjusted predicted probabilities of complicated acute appendicitis (CAA) by neutrophil count, stratified by sex. Predicted probabilities were derived from a multivariable logistic regression model that included an interaction between sex and neutrophil count and was adjusted for age, diabetes, obesity, and hypertension. Points represent individual adjusted predicted probabilities, illustrating population heterogeneity, solid lines represent model-estimated marginal predicted probabilities across the observed range of neutrophil counts, and shaded areas indicate 95% confidence intervals.

Analysis of the interaction between sex and neutrophil count was performed using model-estimated marginal predicted probabilities derived from logistic regression models that included interaction terms, with men as the reference category. This analysis revealed a sex-specific effect. A significant interaction was found between sex and neutrophil count (OR = 0.919 (0.872-0.969), *p* = 0.002), indicating that the association between neutrophils and CAA was stronger (or more positive) in women than in men (Figure 2).

**Figure 3:**
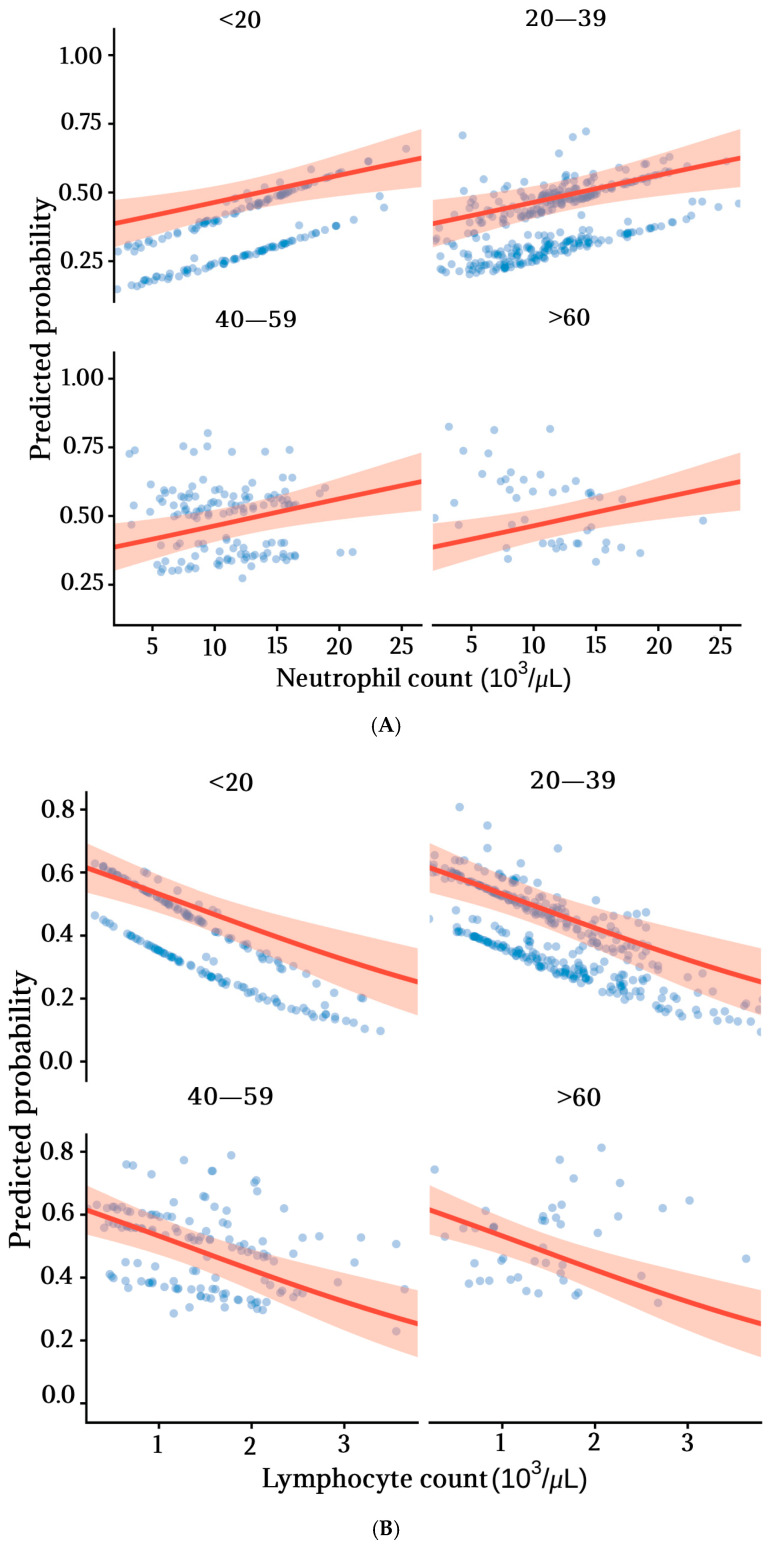
Adjusted predicted probabilities of complicated acute appendicitis (CAA) according to leukocyte subpopulation counts, stratified by age group. Predicted probabilities were derived from multivariable logistic regression models that included interactions between age and leukocyte subpopulation counts and were adjusted for sex, diabetes, obesity, and hypertension. To minimize the influence of extreme values, leukocyte counts are displayed across their 1st to 99th percentiles. Points represent individual adjusted predicted probabilities across this range. Solid lines represent marginal predicted probabilities, and shaded areas indicate 95% confidence intervals. (**A**) Neutrophil count stratified by age groups (<20, 2039, 4059, and >60 years). (**B**) Lymphocyte count stratified by age groups (<20, 20–39, 40–59, and >60 years).

Analysis of the interaction between age and neutrophil count also revealed an age-dependent pattern. A negative interaction was observed (β = −0.002; OR = 0.998 (0.997–1.000); *p* = 0.008), indicating that the effect of neutrophils on CAA diminishes with increasing age. In patients under 20 years of age, higher neutrophil counts were associated with a greater predicted probability of CAA; this association persisted, although attenuated, in the 20–39-year age group. Among patients aged 40 years and older, the association was minimal. In contrast, probability analysis indicated that higher lymphocyte counts were associated with a lower predicted probability of CAA in younger patients, an effect that diminished with advancing age (Figure 3).

These findings suggest that while neutrophil and lymphocyte counts are associated with complicated acute appendicitis, these associations vary by both sex and age.

## 4. Discussion

Acute appendicitis (AA) is a leading cause of abdominal surgery and can occur at any age. The highest incidence is during the second decade of life [32]. Male sex has been associated with a higher prevalence of complicated acute appendicitis (CAA) in both pediatric and adult populations [33]. Consistent with previous reports, in our study, acute appendicitis was more frequent among young adults [34]. CAA, however, was observed more commonly in men [6]. These findings prompted stratified analysis by sex and age. Stratified analysis by sex revealed differences in the proportion of CAA between men and women under 60 years. This pattern was not seen among adults aged 60 years or older.

Previous studies have examined comorbidities, clinical signs, symptoms, and ultrasound imaging findings from a sex-comparative perspective [6]. In our study, we integrate these variables and expand the analysis by also reporting complete blood count parameters and systemic inflammatory indices. This combined approach enables a more comprehensive comparative analysis of men and women. Specifically, we identified differences in neutrophil, lymphocyte, monocyte, and platelet counts, as well as derived markers such as NLR, MLR, and SII. Notably, men had higher neutrophil and monocyte counts, while women showed higher lymphocyte counts. As this was a retrospective observational study, the findings should be interpreted as descriptive associations rather than causal relationships.

The analysis showed that sex and age were each independently associated with CAA, confirming previous research [9,35]. Immune cells—including lymphocytes, monocytes, and platelets—were also associated with CAA: higher monocyte and platelet counts were positively associated with CAA. Reduced lymphocyte counts were generally associated with a higher probability of CAA. These findings match earlier reports showing elevated neutrophil-to-lymphocyte ratios and decreased lymphocyte counts in CAA patients [36,37]. Notably, these associations remained statistically significant after adjustment for sex, age, and comorbidities. Thus, specific immune cell parameters may serve as potential markers of CAA, pending prospective validation.

Interaction analyses with CAA revealed that neutrophil and lymphocyte counts were the variables whose associations with CAA were significantly modified by demographic factors, though in different ways.

Neutrophilia is widely recognized as a marker of acute inflammation and disease severity in various clinical contexts [38]. However, no overall association between neutrophil count and CAA was observed when modeled as a main effect. This apparent discrepancy can be explained by effect modification. The association between neutrophil count and CAA differs in both magnitude and direction across demographic strata. Specifically, a significant association was observed (OR = 0.919, *p* = 0.002 for the interaction term), indicating that the association between neutrophils and CAA was stronger (or more positive) in women than in men. In addition, a negative interaction with age was observed (β = −0.002, *p* = 0.008), indicating that the effect of neutrophils decreases with increasing age. In contrast, no significant associations were identified in men or in analyses that did not account for these interactions. Such heterogeneity may attenuate or nullify the average effect when estimated across the entire population, resulting in a non-significant main effect.

Regarding lymphocytes, a significant interaction with age was also identified (OR = 1.016, *p* = 0.002). This positive interaction indicates that the association between lymphocyte counts and CAA varies across the age spectrum: the protective effect observed in younger patients tends to diminish with age. In contrast, no evidence of heterogeneity in the associations between monocytes or platelets and CAA by sex or age was identified.

Overall, these findings support the presence of demographic heterogeneity in the associations between neutrophil and lymphocyte counts and CAA in the studied population [22,39].

This study has several strengths. First, analyzing a large sample of AA patients enabled robust evaluation of the association between peripheral immune cell counts and CAA. Second, progressively adjusted logistic regression with interaction terms allowed exploration of potential effect modifiers, including age and biological sex, which remain insufficiently examined in the literature. Third, using widely available hematological parameters enhances clinical applicability. Additionally, internal consistency of the results supports the stability of the observed associations.

Several limitations must be acknowledged. Inflammatory markers and disease severity may vary according to symptom duration, causative microorganisms, prehospital treatment, and socioeconomic factors [40,41,42]. Residual confounding cannot be ruled out. Among these variables, time since symptom onset has been most extensively studied and could represent a particularly relevant confounder in acute appendicitis [43,44]. It may influence both the likelihood of complications and the levels of inflammatory biomarkers at admission. However, information regarding the interval between symptom onset and hospital arrival was not systematically available for all patients. This precluded its inclusion in the analyses. Evidence regarding potential sex differences in symptom duration and time to medical attention is heterogeneous and inconclusive [45,46]. It is important to emphasize that the primary objective of this study was to evaluate the associations among sex, age, and preoperative inflammatory cell counts in patients with complicated acute appendicitis. Accordingly, analyses were based on cross-sectional data describing differences between patients with CAA and uncomplicated acute appendicitis (UAA) treated at our institution. An implicit analytical assumption is that the observed associations between sex, age, and cell counts are comparable among patients at different stages of disease progression. This assumption cannot be verified with the available data. Thus, inflammatory profiles and associations should be interpreted with caution.

A further limitation of the retrospective design is the inability to control variables such as immune function testing. Specific inflammatory markers, including C-reactive protein, were unavailable. Including these could clarify or strengthen findings on immune profiles in CAA.

The small number of individuals aged 60 years or older (*n* = 15 women and *n* = 14 men in the CAA group) resulted in limited data for some stratified interaction models. This led to unstable estimates and wide confidence intervals. In certain analyses, we excluded this subgroup to avoid overparameterization and to ensure model convergence and stability. While this approach reduced precision and limited the ability to draw reliable conclusions for older adults, it did not affect the main associations in the overall models. Because age was modeled as a continuous variable in the primary analyses, systematic bias in the overall estimates is unlikely.

This single-center study may not reflect other settings or populations, or healthcare practices, such as diurnal variability in sample collection. Most patients had open appendectomy; laparoscopy was less common, which could affect comparisons with centers that favor laparoscopic surgery [46]. No in-hospital deaths occurred, consistent with low mortality in similar studies [34], but the absence of events limited evaluation of mortality-related factors. Analyses used preoperative admission data, so the surgical approach is unlikely to have influenced hematological profiles. Variation in blood draw timing may have caused diurnal changes in hematologic indices, increasing random variability. Multicenter studies with other context cases are needed to confirm these findings.

No differences in ultrasound detection appeared between men and women. This may relate to the low 4% identification rate of appendiceal and periappendiceal findings in our population. This limited performance matches earlier reports of low positive predictive value for some findings, such as appendicoliths. The operator-dependent nature of ultrasound is another limitation [47]. Thus, ultrasound should be considered a complementary tool, used alongside clinical parameters and biomarkers, which may contribute to improved diagnostic accuracy [48]

In our analyses, sex and age were relevant in both adjustment and interaction models, indicating heterogeneity in the associations between cell counts and CAA according to these variables. This highlights the need to consistently account for sex and age when interpreting hematological markers. Considering these factors may improve diagnostic stratification. Including sex in scoring systems such as RIPASA supports this approach and underscores the importance of demographic variables in evaluating heterogeneous inflammatory diseases such as acute appendicitis [8,49].

## 5. Conclusions

Males exhibit a higher prevalence of CAA and a more pronounced inflammatory profile, as indicated by circulating immune cell counts. Sex, age, and immune cell concentrations are independently associated with CAA, with evidence of heterogeneity across demographic strata. In particular, the associations between lymphocyte and neutrophil counts and CAA vary by age and sex. These findings may inform future risk-stratification strategies.

## Figures and Tables

**Table 1:** Comparison of all preoperative factors between male and female appendicitis groups.

Variable	Global AA (*n* = 708)			UAA (*n* = 411)			CAA (*n* = 297)		
	Female	Male	*p*	Female	Male	*p-FDR*	Female	Male	*p*
Age (continuous)	30 (21–43)	27 (21–39)	0.1975	28 (20–40)	26 (21–38)	0.4487	33 (21–48)	29 (20.50–41.50)	0.1187
<20	68 (46.26%)	79 (53.74%)	0.7736	51 (57.30%)	38 (42.70%)	0.0180 (*)	17 (29.31%)	41 (70.69%)	0.4903
20–39	178 (47.98%)	193 (52.02%)	0.7131	124 (54.86%)	102 (45.14%)	0.7491	54 (37.24%)	91 (62.76%)	0.7080
40–59	78 (55.71%)	62 (44.29%)	0.4855	48 (64.00%)	27 (36.00%)	0.5723	30 (47.37%)	35 (52.63%)	0.5829
>60	26 (52%)	24 (48%)	0.7709	11 (52.38%)	10 (47.62%)	0.7676	15 (51.72%)	14 (48.28%)	0.3146
Self-medication (steroids)	56 (16%)	70 (19.55%)	0.6495	129 (55.12%)	88 (49.71%)	0.6383	83 (71.55%)	110 (60.77%)	0.3833
Self-medication with antibiotics	212 (60.57%)	198 (55.30%)	0.5853	23 (9.82%)	26 (14.68%)	0.0480 (*)	33 (28.44%)	44 (24.30%)	0.6570
Comorbidities									
Diabetes	27 (7.7%)	18 (5.02%)	0.6128	10 (4.27%)	6 (3.38%)	0.7605	17 (14.65%)	12 (6.62%)	0.2300
Obesity	54 (15.42%)	58 (16.20%)	0.7149	36 (15.84%)	22 (12.42%)	0.7627	18 (15.51%)	36 (19.88%)	0.7566
Hypertension	13 (3.71%)	10 (2.79%)	0.6830	6 (2.56%)	7 (3.38%)	0.6710	7 (6.03%)	3 (1.65%)	0.3097
Signs and symptoms									
Anorexia	231 (66%)	246 (68.71%)	0.6616	158 (67.52%)	115 (64.97%)	0.7198	73 (62.93%)	131 (72.37%)	0.5208
Migration of pain	272 (77.71%)	284 (79.32%)	0.7208	180 (76.90%)	139 (78.53%)	0.7762	92 (79.31%)	145 (80.11%)	0.9125
Fever greater than 38 °C	128 (36.57%)	146 (40.78%)	0.6524	78 (33.33%)	59 (33.33%)	0.9999	50 (43.10%)	87 (48.06%)	0.7548
Nausea/Vomiting	300 (85.71%)	299 (83.51%)	0.6975	207 (88.46%)	148 (83.61%)	0.5512	93 (80.17%)	151 (83.42%)	0.6785
Right lower quadrant pain	345 (98.57%)	350 (97.76%)	0.6882	230 (98.29%)	172 (97.17%)	0.6500	115 (99.13%)	178 (98.34%)	0.7171
Right iliac fossa rebound (Blumberg’s sign)	301 (86%)	297 (82.96)	0.6610	197 (84.18%)	141 (79.66)	0.6392	104 (89.65%)	156 (86.18%)	0.7808
Laboratory values									
Leucocytes (10^3^/µL)	13.63 (10.4–17.1)	14.50 (11.32–17.57)	0.1113	12.69 (10.1–16.3)	14.44 (11.1–17.7)	0.0576	14.58 (11.7–17.9)	14.57 (11.4–17.3)	0.7567
Neutrophils (10^3^/µL)	10.92 (7.6–14.4)	11.99 (9.070–14.91)	0.0321 (*)	10.37 (6.9–13.8)	11.82 (8.7–15.2)	0.0159 (*)	12.44 (9.5–15.6)	12.2 (9.2–14.7)	0.6640
Lymphocytes (10^3^/µL)	1.56 (1.0–2.2)	1.285 (0.8475–1.8)	0.0042 (**)	1.68 (1.1–2.3)	1.43 (0.9–2.0)	0.0052 (**)	1.36 (0.9–1.8)	1.18 (0.7–1.6)	0.0561
Monocytes (10^3^/µL)	0.76 (0.5–1.0)	0.89 (0.64–1.2)	0.0021 (**)	0.74 (0.5–1.0)	0.88 (0.6–1.2)	0.0018 (**)	0.81 (0.6–1.0)	0.9 (0.6–1.2)	0.1924
Platelets (10^3^/µL)	262.0 (223.8–312.5)	242 (199.8–287.0)	0.0014 (**)	260.5 (222.0–305.2)	239.0 (200.5–285.5)	0.0048 (**)	267.0 (228.0–345.2)	247.0 (199.0–293.0)	0.0033 (**)
MPV	10.10 (9.6–10.9)	10.10 (9.500–10.70)	0.3852	10.1 (9.6–10.9)	9.9 (9.5–10.7)	0.1799	10.1 (9.6–10.8)	10.1 (9.5–10.7)	0.8533
aPTT(s)	29.80 (27.10–32.20)	29.40 (27.30–32.33)	0.6077	29.7 (27.075–32.1)	29.0 (27.2–31.3)	0.4390	30.1 (27.1–33.0)	29.4 (27.6–33.1)	0.6536
PT(s)	14.60 (13.80–15.85)	14.80 (14.00–15.93)	0.1695	14.3 (13.5–15.5)	14.3 (13.8–15.5)	0.5316	15.3 (14.3–16.5)	15.2 (14.3–16.7)	0.8844
INR	1.100 (1.1–1.2)	1.100 (1.100–1.200)	0.1194	1.1 (1.0–1.2)	1.10 (1.1–1.2)	0.4613	1.20 (1.1–1.3)	1.20 (1.1–1.3)	0.9012
NLR	7.4 (3.7–13.5)	10.06 (13.16)	0.0010 (**)	6.08 (3.1–11.4)	8.4 (4.5–15.3)	0.0021 (**)	9.36 (5.9–14.7)	10.58 (6.4–16.9)	0.3147
PLR	173.8 (118.1–256.5)	201.4 (168.7)	0.0270 (*)	155.97 (112.3–224.8)	184.02 (117.1–260.0)	0.1226	225.30 (149.2–305.4)	212.5 (144.1–327.8)	0.8664
MLR	0.48 (0.3–0.7)	0.7069 (0.6536)	0.0008 (***)	0.42 (0.2–0.7)	0.63 (0.3–1.0)	0.0007 (***)	0.62 (0.3–0.9)	0.76 (0.5–1.1)	0.0032 (**)
SII	1914 (1004–3389)	2299 (1405–3885)	0.0057 (**)	1441.9 (811.1–2944.2)	2167 (1051.4–3674.6)	0.0168 (*)	2640.11 (1606.1–4016.5)	2492.78 (1560.5–4109.8)	0.8493
US finding									
Appendicolith	8 (2.28%)	11 (3.07%)	0.7051	4 (1.70%)	6 (3.38%)	0.6566	4 (3.44%)	5 (2.76%)	0.7892
Periappendicular Plastron	54 (15.42%)	50 (13.96%)	0.7284	27 (11.53%)	20 (11.29%)	0.9723	27 (23.27%)	30 (16.57%)	0.6100
Abscess	14 (4%)	24 (6.70%)	0.5100	4 (1.70%)	3 (1.69%)	1.0078	10 (8.62%)	21 (11.60%)	0.7275
Free fluid in periappendicular cavity	31 (8.85%)	23 (6.42%)	0.6366	13 (5.55%)	1 (0.56%)	0.1140	18 (15.51%)	22 (12.15%)	0.7410
Inconclusive	31 (8.85%)	16 (4.46%)	0.2280	27 (11.53%)	8 (4.51%)	0.1740	4 (3.44%)	8 (4.41%)	0.7677

MPV: mean platelet volume, aPTT: activated partial thromboplastin clotting time, PT: prothrombin time, INR: International Normalized Ratio, NLR: Neutrophil/Lymphocyte Ratio, PLR: Platelet/Lymphocyte Ratio, MLR: Monocyte/Lymphocyte Ratio, SII: Systemic immune-inflammation index, US: ultrasound. Values are presented as frequency (percentage) or median (interquartile range). * *p* < 0.05; ** *p* < 0.01; *** *p* < 0.001. * Statistically significant after false discovery rate (FDR) adjustment; *n* = 708.

**Table 2:** Association between sex, age, and CAA.

	Variables	*p*	OR 95% CI
Model 1	Sex	0.001 (**)	2.06 (1.52–2.79)
	Age (continuous)	0.009 (**)	1.01 (1.00–1.02)
Model 2	Sex	0.000 (***)	2.197 (1.610–2.999)
	Age(continuous)	0.031 (*)	1.017 (1.002–1.033)
	Sex + Age (continuous)	0.318	0.990 (0.969–1.010)

Model 1: Bivariate model, Model 2 adjusted for obesity, hypertension, and diabetes. Sex = Male; * *p* < 0.05, ** *p* < 0.01, *** *p* < 0.001; *n* = 708.

**Table 3:** Association between inflammatory cells and complicated acute appendicitis.

	Variables	*p*	OR 95% CI
Model 1	Neutrophils	0.371	1.013 (0.985–1.041)
	Lymphocytes	0.000 (***)	0.736 (0.576–0.941)
	Monocytes	0.037 (*)	1.495 (1.024–2.185)
	Platelets	0.014 (*)	1.003 (1.001–1.005)
Model 2	Neutrophils	0.421	1.012 (0.983–1.041)
	Lymphocytes	0.000 (***)	0.652 (0.522–0.814)
	Monocytes	0.036 (*)	1.534 (1.029–2.287)
	Platelets	0.001 (**)	1.003 (1.001–1.006)
Model 3	Neutrophils	0.407	1.013 (0.983–1.043)
	Lymphocytes	0.000 (***)	0.656 (0.524–0.817)
	Monocytes	0.032 (*)	1.554 (1.038–2.326)
	Platelets	0.002 (**)	1.003 (1.001–1.006)

Model 1: unadjusted, Model 2: adjusted for sex and age, Model 3: adjusted for sex, age, diabetes, hypertension, and obesity. * Statistical significance * *p* < 0.05, ** *p* < 0.01, *** *p* < 0.001. *n* = 708.

**Table 4:** Analysis of interaction with age and biological sex in CAA.

	Interaction with Age			Interaction with Sex		
	β	*p*	OR 95%CI	β	*p*	OR 95%CI
Lymphocyte	0.016	0.002 (**)	1.016 (1.006–1.026)	−0.037	0.857	0.963 (0.641–1.448)
Neutrophil	−0.002	0.008 (**)	0.998 (0.997–1.000)	−0.084	0.002 (**)	0.919 (0.872–0.969)

*p*: significance of the interaction term in the fully adjusted logistic regression model. Statistical significance ** *p* < 0.01; *n* = 708.

## Data Availability

The datasets generated during and/or analyzed during the current study can be find in the main text and the Supplementary Materials.

## Supplementary Materials

The following supporting information can be downloaded at: https://www.mdpi.com/article/10.3390/pathophysiology33010022/s1. Supplementary Table S1. Findings unrelated to acute appendicitis. Supplementary Table S2. Unadjusted model.
