# Supplementary material for: Association of Sex, Age, and Inflammatory Cell Counts with Complicated Acute Appendicitis
Source: Pathophysiology. 2026 Mar 14;33(1):22. doi: 10.3390/pathophysiology33010022 (PMC13028704; doi:10.3390/pathophysiology33010022)
Supplement: Supplementary file 1 [file pathophysiology-33-00022-s001.zip › pathophysiology-4110702-supplementary.pdf]

# Supplementary Tables

Table S1. Findings unrelated to acute appendicitis.

| Female (n=20) | Male (n=16) |
| --- | --- |
| Ovarian cyst (8) | Hernia (2) |
| Right tubal abscess (2) | Liver abscess (1) |
| Diverticulitis (2) | Diverticulitis (4) |
| Salpingitis (1) | Traumatic bladder injury (1) |
| Piosalpinx (2) | Right ureteral lithiasis (1) |
| Right renal ectasia (1) | Right orchiepididymitis (1) |
| Rectal tumour (1) | Urolithiasis (1) |
| Urinary tract infection (1) | Not reported (5) |
| Pelviperitonitis (1) | |
| Pregnancy (1) | |

Table S2. Unadjusted model.

| | Sex | | Age | |
| --- | --- | --- | --- | --- |
| **Variables** | **Model 1** | **Model 2** | **Model 1** | **Model 2** |
| Neutrophil | 0.003(.926(0.88-0.97)) | 0.002(0.919(0.87-0.96)) | 0.013(0.998(0.997-1.00)) | 0.047(.998(.996-1.00)) |
| Lymphocytes | 0.986(0.996(0.664-1.496) | 0.857(.963(.641-1.448) | 0.001(1.016-1.026) | 0.002(1.016(1.006-1.026)) |
| Monocytes | 0.106(0.588(0.309-1.119)) | 0.060(.532(0.276-1.026)) | 0.70(.995(0.971-1.020)) | 0.758(0.996(0.972-1.021)) |
| Platelets | 0.208(0.997(0.993-1.001)) | 0.191(.997(0.993-1.001)) | 0.503(1.0(1.0-1.0)) | 0.492(1.0(1-1)) |
